# Correction: La Sala et al. New Fast Acting Glucagon for Recovery from Hypoglycemia, a Life-Threatening Situation: Nasal Powder and Injected Stable Solutions. *Int. J. Mol. Sci.* 2021, 22, 10643

**DOI:** 10.3390/ijms24065625

**Published:** 2023-03-15

**Authors:** Lucia La Sala, Antonio E. Pontiroli

**Affiliations:** 1 IRCCS MultiMedica, Lab of Diabetology and Dysmetabolic Disease, PST Via Fantoli, 16/15, 20138 Milan, Italy; 2 Dipartimento di Scienze della Salute, Università degli Studi di Milano, 20122 Milan, Italy; antonio.pontiroli@unimi.it

The authors would like to make corrections to the reference citations in the original article [1]. The author mistakenly deleted “reference 45” [2] during proofreading, adjusted some reference order, and renewed the original “reference 81 and 82” [3,4]. With this change, the order of some reference citations has been adjusted correspondingly. The corrected version of this literature review addresses errors in the numbering of citations that were introduced during the proofreading stage of submission.

The authors apologize for any inconvenience caused and state that the scientific conclusions are unaffected. This correction was approved by the Academic Editor. The original publication has also been updated.
